# Risk Factors for Dysphagia in Patients Hospitalized with COVID-19

**DOI:** 10.1007/s00455-022-10518-1

**Published:** 2022-09-15

**Authors:** Anna Holdiman, Nicole Rogus-Pulia, Michael S. Pulia, Lily Stalter, Susan L. Thibeault

**Affiliations:** 1 Division of Otolaryngology, Department of Surgery, UW-Madison, 5103 WIMR, 1111 Highland Ave., Madison, WI 53705 USA; 2 Division of Geriatrics and Gerontology, Department of Medicine, UW-Madison, Madison, WI USA; 3 Department of Emergency Medicine, UW-Madison, Madison, WI USA; 4 Department of Surgery, University of Wisconsin School of Medicine and Public Health, UW-Madison, Madison, WI USA

**Keywords:** Dysphagia, COVID-19, Pneumonia, ARDS

## Abstract

Patients hospitalized with COVID-19 may be at risk for dysphagia and vulnerable to associated consequences. We investigated predictors for dysphagia and its severity in a cohort of patients hospitalized with COVID-19 at a single hospital center. A large level I trauma center database was queried for all patients hospitalized with COVID-19. Demographics, medical information associated with COVID-19, specific to dysphagia, and interventions were collected. 947 patients with confirmed COVID-19 met the criteria. 118 (12%) were seen for a swallow evaluation. Individuals referred for evaluation were significantly older, had a lower BMI, more severe COVID-19, and higher rates of intubation, pneumonia, mechanical ventilation, tracheostomy placements, prone positioning, and ARDS. Pneumonia (OR 3.57, *p* = 0.004), ARDS (OR 3.57, *p* = 0.029), prone positioning (OR 3.99, *p* = 0.036), ventilation (OR 4.01, *p* = 0.006), and intubation (OR 4.75, *p* = 0.007) were significant risk factors for dysphagia. Older patients were more likely to have more severe dysphagia such that for every 1-year increase in age, the odds of severe dysphagia were 1.04 times greater (OR 1.04, *p* = 0.028). Patients hospitalized with COVID-19 are at risk for dysphagia. We show predictive variables that should be considered when referring COVID-19 patients for dysphagia services to reduce time to intervention/evaluation.

## Introduction

Coronavirus Disease 2019 (COVID-19) has affected over 464 million people and led to the death of over 6 million individuals worldwide as of March 2022 [1]. This virus can cause multi-organ dysfunction resulting in significant morbidity and mortality [2]. Swallowing disorders, known as oropharyngeal dysphagia, are not yet well understood in those hospitalized with COVID-19 sequelae [3–5]. Research has shown that COVID-19 can reduce lung function, thus altering the intricate relationship between swallowing and breathing coordination [5, 6]. Disruption or incoordination of respiratory-swallow patterning increases the risk for aspiration (entry of food/liquid into the lungs) that can lead to pneumonia [5, 7, 8]. Furthermore, due to the nature of the respiratory infection, patients with COVID-19 may experience pulmonary compromise resulting in hypoxic respiratory failure or acute respiratory distress syndrome (ARDS) and ultimately may require endotracheal intubation [5, 7, 9–13]. Prone positioning for intubated patients is a treatment to help patients reach or maintain adequate oxygen saturation levels [5, 14]. However, previous research has speculated that this position may increase the chance of aspiration of secretions, limit the ability for nurses to perform oral care, and potentially could cause laryngeal injury with the movement, dislodgement of the endotracheal tube or can lead to airway obstruction [4, 5, 14, 15]. Pathogenic bacteria and dental plaque colonize in the oral flora of intubated patients, which can lead to infection, pulmonary compromise, ventilator-associated pneumonia, greater length of stay in the ICU, and even death [5, 16–18]. Lastly, post-intubation dysphagia is reportedly prevalent in adults recovering from COVID [19]. These conditions have been implicated in increased risk for the development of dysphagia and its consequences in hospitalized patients with and without COVID-19 [7, 15, 20].

Empirical research and peer-reviewed guidelines are emerging for the care of the COVID-19 patient with dysphagia. Yet, a paucity of research exists to help inform practice patterns and standards of care for dysphagia management amidst this global pandemic [4, 5, 21]. Moreover, the evolving understanding of COVID-19 and restrictive hospital policies related to clinical and instrumental swallowing assessments have challenged dysphagia provider’s workflows and practice patterns [3, 4, 22]. Studies published to date include small patient numbers and have not considered the severity of dysphagia when evaluating predictors of dysphagia in COVID-19 patients. The current study aims to expand on this paucity in the literature by examining risk factors for dysphagia development in a larger group of patients hospitalized with COVID-19 at a single hospital center. We identify risk factors that may predict dysphagia. Improved identification of COVID-19 patients at risk for dysphagia should reduce time to evaluation or intervention, thereby improving patient outcomes.

## Methods

### Study Population

A large level I trauma center’s COVID-19 database was utilized after approval from the Institutional Review Board. This database included select electronic health record (EHR) based data elements for all individuals 18 years or older, hospitalized with confirmed COVID-19 from March 2020 to February 2021. Individuals under 18, with esophageal dysphagia only, or COVID-19 before hospital admission, were excluded via manual chart review. Manual electronic medical record (EMR) abstraction was completed to ensure that patients met inclusion and exclusion criteria and to ascertain variables used to calculate COVID-19 severity, International Dysphagia Diet Standardization (IDDS), and Dysphagia Outcome and Severity Scale (DOSS) scores [23, 24]. Data were stored within an excel spreadsheet. An electronic data abstraction form was utilized, along with a secondary review of all key variables. All IDDSI-FDS and DOSS scores were derived from a chart review of the first author and a separate blinded rater. Medical records with missing data were excluded from this study.

### Variables

Demographic data collected included gender, age, sex, race (white and non-white), and ethnicity (Hispanic or non-Hispanic). Data included from the Institute for Clinical and Translational Research (ICTR) database were body mass index (BMI), history of dysphagia (yes/no), days COVID-19 positive before admission, ventilation which was defined as noninvasive positive-pressure (yes/no), length on ventilation (days), intubation (yes/no), length of intubation (days), number of intubations, tracheostomy (yes/no), use of prone positioning (yes/no), presence of pneumonia via International Classification of Disease (ICD) 10 code (yes/no), presence of acute respiratory distress syndrome (ARDS) via ICD 10 code (yes/no), presence of diabetes via ICD 10 code (yes/no), presence of dialysis (yes/no), and evaluation by the Speech-Language Pathology (SLP) Swallow Service (yes/no). Database managers queried EHR to identify individuals with a history of dysphagia using combinations of dysphagia (i.e., dysphagia, oropharyngeal dysphagia, and pharyngeal dysphagia) in addition to the ICD-10 codes. Next, each chart was manually reviewed to exclude patients with esophageal dysphagia or to identify any individuals that may have received a dysphagia diagnosis during their admission with COVID-19. Individuals with self-reported dysphagia were excluded from this study. COVID-19 severity, based on the World Health Organization Clinical Progression Scale, was manually abstracted and used to quantify the severity of COVID-19 (8). This scale measures patient illness from 0 (not infected) to 10 (death) using the following clinical information—level of supplemental oxygen use, presence of bilevel positive airway pressure (BiPAP) ventilation, mechanical ventilation, extracorporeal membrane oxygenation (ECMO), dialysis, and/or use of vasopressors. Individuals hospitalized with COVID-19 infection fall into moderate (4–5), severe (6–9), or deceased (10) categories [25]. The International Dysphagia Diet Standardization Initiative Functional Diet Scale (IDDSI-FDS) (8) was used as a proxy for oropharyngeal dysphagia in patients evaluated by the SLP Swallow Service. The IDDSI-FDS was based upon the level of diet restriction of liquids and solids recommended following the swallow evaluation. The scale ranges from 0 (nothing by mouth) to 8 (thin liquids and regular solids) and provides a numeric score for the intersection of solid and liquids recommendations, with lower numbers indicating more restriction. Scores from 0 to 7 were considered a modified diet and a surrogate for dysphagia; scores of 8 reflected a regular diet and absence of dysphagia [23]. For individuals who received an instrumental swallow study, the Dysphagia Outcome and Severity Scale (DOSS) was calculated. The DOSS is a 7-point scale used to systematically classify the severity of dysphagia, utilizing instrumental evaluation assessment of physiology, specifically oral stage transfer, pharyngeal stage retention, and airway protection. Additionally, the scale uses diet recommendations, independence level, and type of nutrition; the lower the DOSS score, the greater the severity of dysphagia [24].

### Statistical Analysis

Data analysis was performed using SAS software (version 9.4, SAS Institute Inc., Cary, NC). To assess inter-rater reliability, 20% of DOSS scores and 20% of IDDSI-FDS scores were randomly selected to be re-scored by a secondary reviewer. Within each measure, weighted kappa statistics were then calculated. Demographic, disease, and treatment characteristics of COVID-19 in patients with and without a dysphagia evaluation were compared using two-sample *t*-tests for continuous measures and *χ*^2^ tests for categorical factors. To evaluate differences between those with modified and regular diets among those with IDDSI-FDS scores, two-sample *t*-tests were used to investigate patient age and BMI. In contrast, other continuous variables were analyzed using Wilcoxon Rank Sum tests due to violation of the normality assumption within groups. Categorical variables were examined with Fisher’s exact tests. Unadjusted logistic regression models were used to investigate the association of each patient’s characteristics with the odds of a modified diet. Additional investigations were conducted among the subset of individuals who underwent an instrumental swallow study. The dysphagia (DOSS score 1–5) and normal swallow groups were compared using Wilcoxon Rank Sum tests and Fisher’s exact tests for continuous and categorical variables, respectively. Unadjusted logistic regression models were used to examine the odds of dysphagia by each patient characteristic for individuals with instrumental swallow studies. Additional investigations were conducted examining the severity of dysphagia, with severe dysphagia defined as a DOSS score of 1 or 2, mild to moderate dysphagia defined as a DOSS score of 3–5, and normal swallow function defined as a DOSS score of 6 or 7. Ordinal logistic regression was used to examine the association of each patient characteristic with dysphagia severity. Lastly, we used Kruskal–Wallis *H* tests to examine differences in age and intubation length by pandemic quarter. We used *χ*^2^ tests to investigate differences in the proportion of individuals on ventilation and intubation during each quarter, as well as COVID severity by quarter. A patient’s pandemic quarter was determined by their date of admission. A *p*-value < 0.05 was used for statistical significance.

## Results

From March 2020 to February 2021, 947 patients with confirmed, symptomatic COVID-19 were admitted to a large level I trauma medical center (Table 1). Of those patients, 117 (12%) were seen by the SLP Swallow Service. Patients who were referred for swallow evaluations (64.7 years SD ± 17.4) were significantly older than those without a swallow evaluation referral (59.9 years ± 17.5) (*p* = 0.006) with lower BMI (*p* = 0.001). Patients seen by Swallow Service had greater COVID-19 severity scores (*p* < 0.001), and more pneumonia diagnoses (*p* < 0.001). Further significant differences included higher rates of intubation (*p* < 0.001), mechanical ventilation (*p* < 0.001), tracheostomy placements (*p* < 0.001), prone positioning (*p* < 0.001), and ARDS (*p* < 0.001) in those referred for a swallow evaluation. No significant differences were found based on referral for swallow evaluation for the following variables: gender, race, ethnic group, history of dysphagia, diabetes, receiving dialysis, days COVID-19 positive before admission, or the nu number of days ventilated or intubated.

**Table 1:** Comparison of patients with vs. without swallow consults

	Total population *n* = 947	No swallow consult *n* = 829	Swallow consult *N* = 118	*p* value
Gender				0.850
Male	522 (55.12%)	456 (55%)	66 (56%)	
Female	425 (44.8%)	373 (45%)	52 (544%)	
Age (years)		59.9 (± 17.5)	64.7 (± 17.4)	< 0.006*
Race				0.545
White	779 (84.95%)	686 (85.22%)	93 (83.03%)	
Non-white	138 (15.05%)	119 (14.78%)	19 (16.97%)	
Ethnic group				0.502
Hispanic/Latino	99 (10.68%)	89 (10.03%)	10 (8.85%)	
Not Hispanic/Latino	828(89.32%)	725 (89.07%)	103 (91.15%)	
BMI		31.09(± 9.17)	28.20 (± .55)	0.001
History of Dysphagia				0.266
Yes	211 (22.28)	180 (21.71%)	31 (26.27%)	
No	736 (77.72)	649 (78.29%)	87 (73.73%)	
Days COVID + to admit		3.37 (± 12.84)	2.7 (± 11.3)	0.600
COVID severity				< .0001*
Moderate	626 (66.31%)	582 (70.29%)	44 (37.93%)	
Severe	318 (33.69%)	246 (29.71%)	72 (62.07%)	
Ventilation				< .0001*
Yes	148 (15.63%)	97 (11.70%)	51 (43.22%)	
No	799 (84.37%)	732 (88.30%)	67 (56.78%)	
Ventilation (days)		10.36(± 10.30)	12.01 (± 12.17)	
Intubation				< .0001*
Yes	126 (13.31%)	84 (10.13%)	42 (35.59%)	
No	821 (86.69%)	745 (89.87%)	76 (64.41%)	
Intubation (days)		15.32 (± 26.81)	14.85 (± 17.18)	0.388
Intubation (#)		1.32 (± 0.89)	1.62 (± 1.06)	
Tracheostomy				< .0001*
No	922 (97.36)	815 (98.31%)	107 (90.68%)	
Yes	25 (2.64%)	14 (1.69%)	11 (9.32%)	
Proning				< .0001*
Yes	121 (12.78%)	86 (10.37%)	35 (29.66%)	
No	826 (87.22%)	743 (89.63%)	83 (70.34%)	
Pneumonia				0.0006*
Yes	470 (49.63%)	394 (47.53%)	76 (64.41%)	
No	477 (50.37%)	435 (52.47%)	42 (35.59%)	
ARDS				< .0001*
Yes	112 (11.83%)	753 (90.83%)	36 (30.51%)	
No	835 (88.17%)	76 (9.17%)	82 (69.49%)	
Diabetes				0.3200
Yes	354 (37.38%)	305 (36.79%)	49 (41.43%)	
No	593 (62.62%)	524 (63.21%)	69 (58.47%)	
Dialysis				0.2818
Yes	87 (9.19%)	73 (8.81%)	14 (11.86%)	
No	860 (90.81%)	756 (91.19%)	104 (88.14%)	

*p < 0.05

**Fig. 1:**
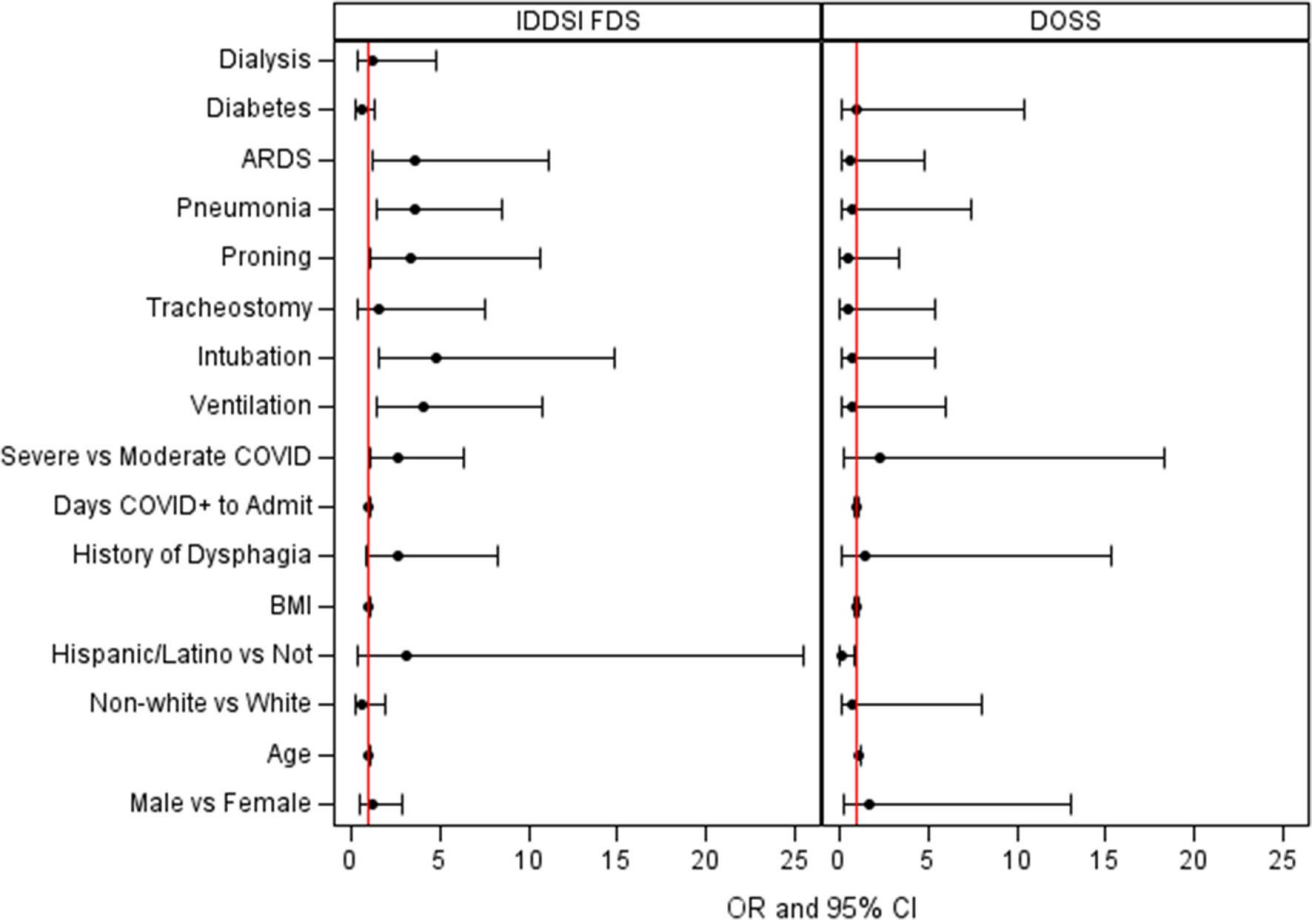
Forest plot of odds ratios and 95% confidence intervals from unadjusted logistic regression models examining the likelihood of a modified diet based on the IDDSI FDS or of dysphagia established from DOSS score

IDDSI-FDS measures (a proxy for swallow function) from the clinical bedside exam for all 117 patients, were found to have high interrater reliability (*K* = 0.91, *p* < 0.001). Patients with dysphagia had significantly higher rates of pneumonia (*p* = 0.007), ARDS (*p* = 0.035), intubation (*p* = 0.004), mechanical ventilation, (*p* = 0.005), severe COVID-19 (*p* = 0.028), and prone positioning (*p* = 0.035) compared to those without dysphagia (Table 2). No significant differences were found for gender, age, race, ethnicity, BMI, history of dysphagia, days of COVID-19 positive to admission, days of ventilation, intubation days, the number of intubations, tracheostomy, diabetes, and dialysis. Unadjusted logistic regression modeling found pneumonia [OR 3.57, 95% CI (3.57, 8.54), *p* = 0.004], ARDS [OR 3.57, 95% CI (1.14, 11.18), *p* = 0.029], moderate COVID-19 [OR 2.69, 95% CI (1.14, 6.37), *p* = 0.025], prone positioning [OR 3.99, 95% CI (1.08, 10.65), *p* = 0.036], ventilation [OR 4.01, 95% CI (1.49, 10.80), *p* = 0.006], and intubation [OR 4.75, 95% (1.52, 14.80), *p* = 0.007] to be significant risk factors for dysphagia (Table 2, Fig. 1).

**Table 2:** IDDSI-FDS bedside swallow evaluation

	Dysphagia *n* = 88	No Dysphagia *n* = 29	*p* value	Odds ratio	95% Confidence Interval	*p* value
Gender			0.6708	1.23	0.53–2.84	0.632
Male	50 (56.82%)	15 (51.72%)				
Female	38 (43.18%)	14 (48.28%)				
Age (years)	65.13 (± 16.62)	62.76 (± 19.47)	0.5257	1.01	0.98–1.03	0.522
Race			0.3967	0.64	0.21–1.90	0.420
White	71 (84.52%)	21 (77.78%)				
Non-white	13 (15.48%)	6 (22.22%)				
Ethnic Group			0.4464	3.08	0.37–25.47	0.297
Hispanic/Latino	9 (10.59%)	1 (3.70%)				
Not Hispanic/Latino	76 (89.41%)	26 (96.30%)				
BMI	27.97 (± 8.85)	28.98 (± 7.80)	0.5257	0.99	0.94–1.04	0.582
History of Dysphagia			0.1399	2.62	0.83–8.28	0.101
Yes	26 (29.55%)	4 (13.79%)				
No	62 (70.45%)	25 (86.21%)				
Days COVID + to admit	3.05 (± 12.77)	1.75 (± 5.05)	0.7900	1.01	0.97–1.07	0.602
COVID severity			0.0275*	2.69	2.24–6.37	0.025*
Moderate	27 (31.40%)	16 (55.17%)				
Severe	59 (68.60%)	13 (44.83%)				
COVID severity			0.1351			
4	5 (5.68%)	3 (10.34%)				
5	20 (22.73%)	11 (37.93%)				
6	13 (14.77%)	5 (17.24%)				
7	6 (6.82%)	1 (3.45%)				
8	2 (2.27%)	2 (6.90%)				
9	42 (47.73%)	7 (24.14%)				
Ventilation			0.0048*	4.01	1.49–10.80	0.006*
Yes	45 (51.14%)	6 (20.69%)				
No	43 (48.86%)	23 (79.31%)				
Ventilation (days)	12.54 (± 12.74)	7.97 (± 5.46)	0.5886			
Intubation			0.0039*	4.75	1.52–14.80	0.007*
Yes	38 (43.18%)	4 (13.79%)				
No	50 (56.82%)	25 (86.12%)				
Intubation (days)	13.99 (14.63)	22.98 (36.00)	0.9488			
Intubation (#)	1.57 (± 1.89)	1 (± 0.67)	0.2756			
Tracheostomy			0.7293	1.54	0.31–7.56	0.597
No	79 (89.77%)	27 (93.10%)				
Yes	9 (10.23%)	2 (6.90%)				
Care level			0.5497			
General	22 (25%)	10 (35.71%)				
ICU	54 (61.36%)	15 (53.57%)		1.64	0.64–4.20	0.305
IMC	12 (13.64%)	3 (10.71%)		1.82	0.43–7.90	0.425
Proning			0.0352*	3.40	1.08–10.65	0.036*
Yes	31 (35.23%)	4 (13.79%)				
No	57 (64.77%)	25 (86.21%)				
Pneumonia			0.0066*	3.57	1.49–8.54	0.004*
Yes	63 (71.59%)	12 (41.38%)				
No	25 (28.41%)	17 (58.62%)				
ARDS			0.0352*	3.57	1.14–11.18	0.029*
Yes	32 (36.36%)	4 (13.79%)				
No	56 (63.64%)	25 (86.21%)				
Diabetes			0.2784	0.59	0.25–1.37	0.2177
Yes	34 (38.64%)	15 (51.72%)				
No	54 (61.36%)	14 (48.28%)				
Dialysis			1.000	1.24	0.32–4.78	0.7568
Yes	11 (12.50%)	3 (10.34%)				
No	77 (87.50%)	26 (89.66%)				

*p < 0.05

Due to restrictions in performing aerosol-generating procedures in our institution during the initial stages of the COVID-19 pandemic, only 41 patients in our cohort completed either a videofluoroscopic swallow study (VFSS) or fiberoptic endoscopic evaluation of swallowing (FEES). DOSS measures calculated to determine dysphagia presence and severity from these exams had high inter-rater reliability (*K* = 0.82, *p* < 0.001). COVID-19 patients who were older were statistically more likely to have dysphagia (*p* = 0.050). No other statistically significant differences were found. Logistic regression analysis found that Hispanic or Latino/a/x patients were less likely to be significantly associated with having a dysphagia diagnosis versus non-Hispanic/Latino/a/x patients [OR 0.091, 95% CI (0.062, 6.302), *p* = 0.04]. Ordinal logistic regression examining the severity of dysphagia found that for every 1-year increase in age, the odds of severe dysphagia were 1.04 times greater [OR 1.04, 95% CI (1.004, 1.081), *p* = 0.028] (Fig. 1). Similarly, the odds of the combined categories of mild/moderate and severe dysphagia versus no dysphagia are 1.04 times greater for each year’s increase in age. No other variables were statistically significant in the ordinal logistic regression.

To determine if there was an influence of the pandemic wave and evolving practice patterns on intubation and mechanical ventilation we assessed these patient characteristics across quarters (Table 3). Nonsignificant differences were measured for number of patients intubated (*p* = 0.760), days of intubation (*p* = 944), ventilation (Y/N) (*p* = 0.099), and days of ventilation (0.298) across the year. A significant difference in age between quarters (*p* < 0.001) was found, with patients with COVID during the second quarter to be significantly younger than those with COVID during the remainder of the year.

**Table 3:** Age, intubation rate and duration by pandemic quarter (PQ)

	Patients (*n*)	Age (years/SD)	Intubation (Y/%)	Intubation (days/SD)	Ventilation (Y/%)	Ventilation (days/SD)
March–May 2020 (PQ1)	86	61.29 (15.20)	13 (15.12)	10.83 (10.52)	20 (23.26)	9.92 (6.72)
June–Aug 2020 (PQ2)	95	53.93 (18.72)	15 (15.79)	13.09 (13.02)	17 (17.89)	9.39 (9.06)
Sept–Nov 2020 (PQ3)	415	60.10 (17.28)	53 (12.77)	12.67 (16.83)	66 (15.90)	12.39 (11.50)
Dec 2020–Feb2021 (PQ4)	351	62.73 (17.67)	43 (12.25)	20.28 (34.56)	45 (12.82)	9.81 (12.31)
*p* value		< .001	0.760	0.944	0.099	0.298

## Discussion

This study aimed to identify predictors of oropharyngeal dysphagia, among individuals suspected to have dysphagia, in a large cohort of patients hospitalized with confirmed COVID-19. Results suggest that pneumonia, ARDS, prone positioning, ventilation, and intubation are significant predictors of dysphagia development. Older patients are at higher risk for severe dysphagia. These predictors should be considered when considering the appropriateness and necessity of a referral to SLPs specializing in diagnosing and treating oropharyngeal dysphagia in the hospitalized COVID-19 population.

The relationship between aging and dysphagia has been well described in the literature. The prevalence of dysphagia increased with age, with an estimated 10–20% of people over 65 years of age demonstrating swallowing problems [26, 27]. Geriatric swallowing function is influenced by several age-related sensory and motor function declines [28]. Patients referred for swallow evaluation in this study were significantly older than those not referred for a swallow evaluation. Results of our clinical and instrumental evaluations are analogous with demonstrating increased risk for the development of dysphagia and more severe dysphagia. These findings are supported by Dawson et al., who report a mean patient age of 67.6 years ± 17.6 in a cohort of patients hospitalized with COVID-19 seen for suspected dysphagia [3]. Further investigation is warranted to characterize dysphagia in this aged population.

In this study, Hispanic or Latino/a/x individuals were less likely to be significantly associated with having a dysphagia diagnosis versus non-Hispanic/Latino/a/x individuals per instrumental swallow study. This finding is inconsistent with current research that suggests that consequences of COVID-19 infections differ by ethnicity, with greater rates of COVID-19 infection and death in Black, Hispanic, and Asian populations [29–31]. Disparities in healthcare access, preexisting comorbidities, inability to limit exposure due to housing arrangements or occupations may influence COVID-19 outcomes between ethnic groups [29, 31]. It is important to note that in our study only forty-one patients received instrumental swallow study evaluation and only five out of the forty-one were Hispanic/Lationo/a/x, thus warranting cautious interpretation of findings.

Patients with COVID-19 are a unique population as the disease progression and sequelae differ significantly between patients. To quantify disease severity, we employed the WHO-Clinical Progression Scale, which focuses on critical characteristics distinguishing disease progression. Individuals seen by swallow service had more severe COVID-19 than those that did not receive a consult. For patients with dysphagia per bedside evaluation, higher rates of more severe COVID-19 and moderate COVID-19 were predictors for dysphagia development. Previous research has not distinguished the severity of COVID-19 via the WHO Clinical Progression Scale among participants [3, 21, 32]. This is most likely due to evolving resources and should be viewed as a context rather than a design limitation.

Pneumonia is a clinical outcome of dysphagia that can lengthen hospital stays, increase mortality, and lead to hospital readmission [33–36]. A disruption in swallowing, a neurologically mediated function, which includes both voluntary and involuntary activity, can result in aspiration-related pneumonia due to pulmonary infiltrates. Patients who are critically ill may have a compromised cough reflex and are therefore more vulnerable to aspiration of oropharyngeal colonization of pathogenic bacteria and secretions [37, 38]. Pneumonia in patients with COVID-19 can be multifactorial in etiology, however, it is a severe complication [39]. We found 71.59% of patients hospitalized with COVID-19 also had pneumonia, which is similar to other studies, reporting 61%, 54.2%, and 82.8% [32, 40, 41]. Furthermore, we found that patients with swallow service consults had higher rates of pneumonia than those without consults, and pneumonia was a predictor of dysphagia based on bedside evaluation. We did not distinguish the origin of the patient’s pneumonia (i.e., community-acquired and aspiration-related) due to constraints within retrospective records review.

ARDS is defined as acute onset of serious lung injury due to hypoxia, inflammation, and pulmonary edema which often results in respiratory failure [42, 43]. A disruption in respiration and swallowing pattern due to ARDS can put patients at increased risk for dysphagia development along with respiratory interventions associated [5]. ARDS is a common consequence of COVID-19 in critically ill patients. We found that patients seen by swallow service and individuals found to have dysphagia per bedside evaluation had higher rates of ARDS. The treatment of ARDS may ultimately require mechanical ventilation or intubation, which often results in dysphagia [5, 7, 9–13]. Prolonged endotracheal intubation is defined as 48 h or longer may result in dysphagia with incidence rates ranging from 3% [44] to 93% [43, 45, 46]. The etiology of dysphagia post-extubation is multifactorial in nature with mechanical causes related to the endotracheal tube and its impact on laryngeal edema, atrophy, reduced sensation, and possible laryngeal injury [47, 48]. Additionally, patients may be combatting delirium, global deconditioning or neuromuscular weakness along with the effects of weaning off sedative medications that may impact swallow function [7, 48, 49]. Current literature surrounding COVID-19 and post-extubation dysphagia is emerging; a recent study by Regan et al. found that 90% of patients required diet modification based on bedside evaluation, with 66% presenting with post-extubation dysphagia [19]. In our study, mechanically ventilated and intubated patients had higher rates of swallow service consults and dysphagia per bedside evaluation. Additionally, mechanical ventilation and intubation were predictors of dysphagia per bedside evaluation. Further research focused on acute and post-acute postextubation dysphagia should be explored due to concern for decreased lung function and possible development of pulmonary fibrosis as a result [7].

Previous research has hypothesized that prone positioning may impact dysphagia and glottic injury. The utilization of positioning may increase the risk for aspiration or secretions while impacting oral care routines and increase the risk for glottic injury due to movement of endotracheal tube associated with changes between supine and prone position [4, 5, 14, 15]. However, no study has demonstrated a causal relationship between prone positioning and increased risk for dysphagia [5, 14]. In our sample population, patients that were proned had higher rates of swallow service consultation and were more likely to have dysphagia based on bedside evaluation. Our research aligns with the limited available literature exploring this relationship. Regan et al. found proning a predictor of oral intake status post-extubation, with 90% requiring diet modification [19]. Additionally, in a recent study, Naunheim et al. noted that 100% of patients with prone positioning demonstrated glottic pathology under laryngoscopy and stroboscopy, although the study was not sufficiently powered statistically to detect differences between individuals proned and unproned [14]. Further studies should delineate the relationship between prone positioning, laryngeal injury, and possible effects on dysphagia.

Some patients who require long-term ventilation or cannot “wean” off respiratory support may require tracheostomy placement [50]. Current research suggests dysphagia can occur in 11–93% due to anatomical location of placement, effect on swallow function due to shared pathway for deglutition and respiration, and overall medical acuity necessitating placement [51]. While emerging rates of tracheostomy placement reported in COVID-19 patients vary significantly from 6 to 61% in our study, the only statistically significant finding was that individuals with a tracheostomy had higher rates of swallow service consultation [50, 52], 53]. There was no statistically significant relationship between tracheostomy and dysphagia diagnosis via bedside or instrumental evaluations. However, only 25 (2.64%) of all COVID-19 patients received a tracheostomy in our study, with only 11 out of the 25 patients seen by swallow service. This is likely due to consensus on tracheostomy placement varying greatly at the start of the pandemic. Tracheostomy placement was often deferred due to the aerosolizing nature of placement procedures in efforts to limit exposure to healthcare workers [25, 52]. As a result, our findings likely reflect our institution's expert consensus, professional organization recommendations, and hospital guidelines/protocols from March 2020 to February 2021 likely leading to fewer placements than current practice patterns.

This study has limitations that warrant discussion. First, it is important to note that we cannot assume individuals that were not consulted for SLP evaluation did not have dysphagia. Our analysis for predicting risk factors for dysphagia was among those with bedside and/or instrumental swallow studies. Thus, our results are looking at risk factors for dysphagia among those suspected of having dysphagia. The nature of retrospective records review reduces the ability to control variables such as the timing of evaluation and volumes or foods used for bedside evaluation. Additionally, our study was influenced by our hospital’s policy to limit aerosol-generating procedures during the early stages of the pandemic. Ninety percent of patients that had instrumental swallow studies were found to have oropharyngeal dysphagia. This is likely due to the triaging and prioritization that only specific patients with a difficult clinical course, suspected laryngeal injury, or no signs of clinical improvement at the bedside were taken for instrumental swallow studies [3]*.* Another limitation of the study is using the IDDSI-FDS as a dysphagia proxy, as some patients may have a modified diet for reasons other than oropharyngeal dysphagia. For example, a patient with impulsive feeding behaviors may have a modified diet versus true oropharyngeal dysphagia. Lastly, as this investigation considered the 1st year of the COVID pandemic from one institution. While we did not find differences in our practice patterns regarding intubation and mechanical ventilation across the year, this may not be the same for all institutions. Future work detailing the entire time period of the pandemic across multiple sites, is necessary to corroborate our findings.

In summary, our results propose a set of predictive variables for the development of oropharyngeal dysphagia, along with identifying potential risk factors for individuals hospitalized with COVID-19. The evolving nature of COVID-19 and the emergence of novel variants requires continued attention to understand the sequelae of this disease process. Further future research should characterize and determine dysphagia profiles in larger, more robust cohorts of patients receiving instrumental evaluations of swallowing. Identification of medically fragile COVID-19 patients at risk of dysphagia development is crucial to provide intervention to improve patient outcomes and mitigate possible associated medical or pulmonary compromise.
